# Does Body Memory Exist? A Review of Models, Approaches and Recent Findings Useful for Neurorehabilitation

**DOI:** 10.3390/brainsci14060542

**Published:** 2024-05-25

**Authors:** Chiara Parma, Federica Doria, Aida Zulueta, Marilisa Boscarino, Luca Giani, Christian Lunetta, Eugenio Agostino Parati, Mario Picozzi, Davide Sattin

**Affiliations:** 1 Istituti Clinici Scientifici Maugeri IRCCS, Health Directorate, Via Camaldoli 64, 20138 Milan, Italy; cparma1@uninsubria.it (C.P.); 62202191@mail.sfu.ac.at (F.D.); 2 PhD. Program, Medicina Clinica e Sperimentale e Medical Humanities, Insubria University, 21100 Varese, Italy; 3 Istituti Clinici Scientifici Maugeri IRCCS, Labion, Via Camaldoli 64, 20138 Milan, Italy; aida.zuluetamorales@icsmaugeri.it; 4 Neurorehabilitation Department, Istituti Clinici Scientifici Maugeri IRCCS, Via Camaldoli 64, 20138 Milan, Italy; marilisa.boscarino@icsmaugeri.it (M.B.); luca.giani@icsmaugeri.it (L.G.); eugenio.parati@icsmaugeri.it (E.A.P.); 5 Amyotrophic Lateral Sclerosis Unit, Neurorehabilitation Department, Istituti Clinici Scientifici Maugeri IRCCS, Via Camaldoli 64, 20138 Milan, Italy; christian.lunetta@icsmaugeri.it; 6 Center for Clinical Ethics, Biotechnology and Life Sciences Department, Insubria University, 21100 Varese, Italy; mario.picozzi@uninsubria.it

**Keywords:** body memory, cognitive rehabilitation, neurorehabilitation, memory, phenomenology

## Abstract

Over the past twenty years, scientific research on body representations has grown significantly, with Body Memory (BM) emerging as a prominent area of interest in neurorehabilitation. Compared to other body representations, BM stands out as one of the most obscure due to the multifaceted nature of the concept of “memory” itself, which includes various aspects (such as implicit vs. explicit, conscious vs. unconscious). The concept of body memory originates from the field of phenomenology and has been developed by research groups studying embodied cognition. In this narrative review, we aim to present compelling evidence from recent studies that explore various definitions and explanatory models of BM. Additionally, we will provide a comprehensive overview of the empirical settings used to examine BM. The results can be categorized into two main areas: (i) how the body influences our memories, and (ii) how memories, in their broadest sense, could generate and/or influence metarepresentations—the ability to reflect on or make inferences about one’s own cognitive representations or those of others. We present studies that emphasize the significance of BM in experimental settings involving patients with neurological and psychiatric disorders, ultimately analyzing these findings from an ontogenic perspective.

## 1. Introduction

Nowadays, neurologists widely agree on the presence of mental representations of the body, which refer to neural coding patterns located in distinct brain regions or a network of brain areas that encode and monitor the body’s status in both time and space [1,2]. This process is continuously updated with information from both bottom-up and top-down sources [3], as well as internal and external stimuli. Body representations go beyond mere metric information, with various models and taxonomies that propose multiple representations of the body [4]. Conditions like phantom limb syndrome and anorexia nervosa highlight the complexity of body representations [5], encompassing aspects such as body schema, body image, body awareness and so on. Furthermore, factors like physical, emotional, and cultural influences, personal experiences, and traumatic events all seem to contribute to the development and maintenance of body representations. Among the body representations, body memory (BM) has captured the interest of various research groups worldwide considering its peculiarity [2,6,7], sharing a fundamental idea that could not exist a present time without a past time. However, the idea of a memory of the body, the same body that could be the place where the memory exists, represents a controversial idea that some research groups have tried to analyze in the last decades. As known, several models on memory have been proposed in cognitive psychology to explain memory functioning. One of the earliest models, proposed by Atkinson and Shiffrin in 1968, known as the multistore model of memory (or the modal model) [8], categorizes memory into three: a sensory register, short-term memory, and long-term memory. Further models have introduced the concepts of working memory, which involves the ability to temporarily store and manipulate information for specific purposes through a multicomponent system [9]. In the meantime, the terms implicit and explicit memory were introduced as components of long-term memory, which is responsible for storing a vast amount of information over an extended period of time [8]. Implicit memories, often unconscious, involve skills, habits, and conditioned responses, while BM in trauma tends to be implicit, manifesting as physiological reactions triggered by stimuli. Explicit memories [10], on the other hand, are consciously accessible and include facts, events, and personal experiences. They are commonly referred to as declarative memoriessince they can be expressed voluntary through communication and language. After that, explicit memory was further divided into episodic and semantic memory, providing important results that highlighted thatthe former pertains to personally experienced events with an autobiographical context, while the latter refers to the conscious and intentional memory of concepts and meanings. In this brief (and incomplete) summary of memory conceptualization, memory is often associated with a form of storage of external objects, words, or other things that can be perceived in the third person perspective, while what refers to our body, as an object of the memory process remains controversial and unique in the scientific literature.

The concept of BM originates from the field of phenomenology, which has a long-standing focus on trauma. Phenomenology mainly focuses on investigating and describing phenomena, the unmediated and instant experiences that form our perception of the world, trying to suspend judgments, interpretations or preconceived notions and accurately describe what emerges in awareness directly. Even BM is conceived with a non-representational character, implicitly sedimenting in our life history and silently influencing our present experience. This memory emerges in specific contexts and becomes evident in moments of hesitation, fatigue, or adaptation to unexpected circumstances, as happened in the case of trauma experiences [11]. Indeed, extensive literature has focused on the concept of “BM” concerning trauma [12,13,14], distinguishing between implicit and explicit memories related to body representations. While BM may not always be explicitly recalled, explicit memories can be impacted by shaping our emotional responses and behavioral patterns, a process that can be more easily observed in the context of trauma [15]. Traumatic experiences are often stored in memory with heightened emotional intensity and sensory detail. The persistence of trauma-related symptoms may be influenced by BM, which perpetuates physiological arousal and reactivates trauma-associated sensations. 

However, there is limited literature dedicated to investigating the role of BM from a neurological perspective, considering evidence from conditions such as stroke or neurodegenerative diseases. BM has gained more attention in the field of embodied cognition research as will be further discussed in the next sections; therefore, in this article, we provide a comprehensive overview of published studies on this representation, analyzing different definitions and theoretical models proposed, as well as the empirical settings used to test them. Moreover, we discuss some empirical research, assessments and implications for rehabilitative approaches of BM, with the lens of embodied cognition. 

## 2. Definitions and Nature of Body Memory

The term body memory (BM) represents all past bodily experiences that are stored (somewhere) and affect our behavior [16]. The nature of these experiences is still unclear, and different authors have proposed various hypotheses. Merleau-Ponty [17] suggested that BM provides us with the bodily know-how, i.e., the knowledge of how to act with or towards a specific part of our body, supporting the idea that bodily awareness is connected to our senses and movements. He explains that our body has two distinct layers [17]: the habit-body (body memory) and the body at the present moment (body representations). Therefore, our body should be understood not just in its own unique and complete experience but also in a general aspect and with the help of an impersonal being. In order to elucidate the functioning of BM, particularly from a neurobiological perspective, BM has been analyzed phenomenologically as a network of predispositions towards the external world. This network consists of interconnected loops anchored within the body, influenced by the body’s habits, movements, and sensory perceptions. Continuously, this network scans the surroundings for resemblances, compatible elements, and scenarios that it can link to, aiming to establish coherence [6]. The body, within this particular framework, functions as a ‘system that seeks meaning’, actively investigating the surroundings for consistency. In every circumstance we come across, we inevitably tap into our body memories, relying on this consistency [6]. Consequently, they encompass not only tactile, motor, proprioceptive, painful, and interoceptive encounters but also the emotions linked to them. Merleau-Ponty affirmed that these memories can be both explicit and implicit, and they influence our life and mental health, especially during significant emotional experiences such as moments of physical well-being or, on the opposite, accidents and traumatic events. While implicit memories [15] are difficult to reflect on consciously and verbalize in everyday life, they still have a significant impact on our behavior and emotions, contributing to shaping the sense of Self and identity through the structures accumulated in BM.

More recently, Fuchs (2012) [15] affirmed that memory does not just refer to remembering past events explicitly, but also includes our implicit dispositions, habits, and skills that influence our present behavior and experience. According to him, implicit memory is formed by the routine movements of the body, creating a connection between us and the world through its intentional behaviors. Fuchs was among the first researchers to define BM, combining the phenomenology approach with psychiatry, differentiating procedural, situational, intercorporeal, incorporative, pain, and traumatic memories (as described later). Several mental health illnesses have been linked to negative body memories that in the right circumstances can be recalled [6]. Gentsch and Kuehn [16] have examined this line of reasoning in order to determine if negative body memories, as stored in implicit memory, contribute to clinical manifestation of BM mechanisms, defined by the authors as persistent bodily symptoms such as trauma, pain, dissociation, and others. As a result, many authors affirm [18] that the body is a multi-sensory “object” that needs the processing and integration of different bodily signals in the premotor, temporoparietal, posterior parietal, and extrastriate cortices. Our experience of the body is not direct; rather, it is mediated by perceptual information and influenced by internal information such as interoception, proprioception, and vestibular input [19,20]. It is recalibrated through stored implicit and explicit body representations [19,20,21], which together constitute BM.

Beyond the phenomenological approach, neuroscience has recently brought little attention to the role of BM in focusing on the concept of body representation, i.e., cognitive structures that have the role of tracing and coding the state of the body [22]. In cognitive psychology and clinical neuroscience, multisensory body experiences are also used to ground BM [23,24]. As Riva [2] (p. 253) argues, BM is a concept that “*directly connects the experience of the body with intentional development of the self*”. Therefore, it can be seen as a significant bridge between cognitive approaches that address bodily experiences and volitional approaches that emphasize the sense of self and its intentionality.

## 3. Explicative Models of Body Memory

Recently, *phenomenology* has correlated bodily memory to implicit memory [25,26,27], which contains all past bodily experiences that re-emerge in the present situation, improving the quality rather than the content of the experience. Since we are inserted in a biological, cultural, and intersubjective context, bodily memory is linked to embodiment; it is, in fact, anatomically linked to the shape of our bodies, their possibilities of action and their embeddedness in the environment. BM is therefore a situated and embodied phenomenon that contributes to making us unique as people. To date, there are several theories on BM born from phenomenology [15,28,29]. In this perspective, BM includes not only explicit memories of our past but also dispositions, skills and habits acquired during our embodied experiences (mainly in early childhood) which, implicitly, influence present and future experiences and behavior and that can be modified throughout life [6]. The term BM, therefore, refers to all the implicit knowledge, skills, and dispositions that structure and guide our everyday being-in-the-world without the need to explicitly remember what we have done, how we can do it, or to anticipate what we want to do.

BM has been described differently in *cognitive psychology*, for example, as procedural or implicit memory, distinguishing it from explicit or declarative memory (e.g., [26]). In support of the existence of multiple memory systems, research on amnesic patients (e.g., with global amnesic syndrome) has highlighted that although they are unable to retain new memories and learn new material explicitly, they are still capable of learning simple motor tasks, as their implicit memory is preserved.

O’Shaughnessy [30] suggests that BM can be understood as a long-term body representation. The idea of the existence of a long-term body representation is based on the presence of a common content in all short-term body images over time. This is reinforced by the fact that while proprioception’s content is spatial and postural sensations that contribute to developing proprioception, it cannot be the original source of spatial content in proprioception. The dynamic nature of proprioception, which can easily change depending on the postural and bodily sensations experienced, would suggest the existence of a long-term memory that provides a common spatial content. Support for this statement comes from individuals with phantom limb sensation who are born without limbs [31] or who undergo limb amputation at a young age [32].

In this scenario, our BM, partly determined innately and partly malleable by coordinated experience, would allow the construction of different short-term body images that provide real-time information on posture and body position [30]. In this perspective, BM is responsible for the constant implementation of our sedimented past experience rather than making a past experience explicitly present. In particular, BM allows us to learn about perceptual and experiential patterns that mediate familiarity and continuity with events and with our bodily capabilities. Learning with the body occurs through the passage from what we have done explicitly to what passes into implicit and unconscious knowledge.

Other authors, considered BM as the basis of the development of individual styles of perception and movement, and more generally, of ways in which to experience the world [11]. We store our memories in our neural networks, as well as in the activation patterns of muscle fibers. This translates BM as a very important mechanism for organizing things along a continuum of movement, from reflexive movement to motor planning, driving states, and very spontaneous and creative movements. Therefore, BM is strongly a Gestalt between the brain and the rest of the body, while for autobiographical memory, there is not much evidence outside the hippocampus [11,33].

Phenomenology distinguishes different forms of BM in particular, making the distinction between implicit and non-conscious memory (knowing how) and memory connected to explicit and conscious remembering (knowing that) [6]. Unlike cognitive psychology, which defines implicit memory as procedural memory, Fuchs further developed the construct and proposed a detailed descriptive taxonomy with five forms of BM based on the phenomenological tradition of Bergson [28], Merleau-Ponty [17] and Casey [29]. See Table 1 for a description of the five forms of BM defined by Fuchs.

Intercorporeality is the primal form of mutual bodily understanding, which happens in interaction (see Merleau-Ponty [36]). The intercorporeal memory, as patterns of interactions or implicit relational styles, refers to “embodied personality structures”, as called by Fuchs [37], which are important for both psychopathology and psychotherapy. The traumatized person’s intercorporeal memory changed profoundly, in fact, they feel constantly vulnerable, always on guard, and their trust in the world has been irreparably broken. Research on post-traumatic stress disorder teaches us about the specific significance and the rules of this memory system [12].

Kolter et al. [38] conducted an empirical study to examine Fuchs’s BM taxonomy [12]. They utilized content analysis of interviews and questionnaire data to determine if the taxonomy adequately covers all the significant aspects that individuals associate with the concept of BM. They found that all of Fuchs’ categories emerged in participants’ narratives. They saw that regarding the definition of BM, it was striking that participants more often mentioned negative or painful experiences than positive ones. Given the responses of the participants, Koch and colleagues [39] saw that it is possible to differentiate situational BM into sense modalities but it is also possible to narrow its definition down to spatial memory for interior and exterior spaces. Based on the content analysis, Koch et al. [39] extended the taxonomy of Fuchs into a model that includes a “closer to the core layer” of the four basic types of body memories: procedural, intercorporeal, incorporative, and traumatic; the more external layer of situational BM, which is a broader category than the others, and was subdivided into memory forms actualized through the different senses (e.g., auditory, haptic, olfactory, gustatory, and kinaesthetic memory). Moreover, the category of traumatic memory was divided into two subcategories that were always clearly distinct [39]: (a) body memories related to physical pain (pain BM), and (b) body memories related to psychological trauma. Claparede [40], for example, described an amnesic patient who had noexplicit recollection of a previous meeting and was not able to recognize him. One day, he shook hands with the patient while hiding a tack. The patient, feeling the pain, quickly withdrew her hand, and the next day refused to greet him, although she could not explain why. The experience of pain remained imprinted in her body’s memory. The memory of pain may also find expression in psychosomatic illnesses.

More recently, Riva [2] tried to provide a model to better elucidate the neural and psychological bases of BM and its relation with the self, identifying body representations that converged in the body matrix. These include the Sentient Body (Minimal Selfhood), the Spatial Body (Self Location), the Active Body (Agency), the Personal Body (Whole Body Ownership and Me); the Objectified Body (Objectified Self and Mine), and the Social Body (Body Satisfaction and Ideal Me) [2] as reported in the Table 2. The first three body representations (Sentient Body, Spatial Body, Active Body) relate to the subjective experience of the body as a point of reference and are associated with the body schema. On the other hand, the three others (Personal Body, Objectified Body, Social Body) involve a reflective understanding of the body from an objective viewpoint, particularly about how it is perceived by others, thus connecting more closely to the concept of body image. Throughout the lifespan, BM develops ontogenetically, and it is strictly related to the development of the self, as the body is the physical component of the self. A study [41] in the cognitive and neuroimaging domain demonstrates that the body is the most important object in the world, with specific neural areas responsible for its processing.

Another interesting model for BM was derived from the initial idea of Barsalou, who elaborated on Glenberg’s viewpoint, developing a theory of knowledge called the perceptual symbol system (PSS) [42,43]. Based on the PSS model, the association areas in the brain capture bottom-up activation patterns occurring in the sensorimotor areas through a perceptual memory system. In turn, the association areas reactivate the sensorimotor areas to create perceptual symbols through an opposite top-down process. Thus, memory could be considered a sensory-motor encoding [44]. According to this perspective, cognition “*is strongly influenced by the body*” [45] (p. 573) and memory is subject to body manipulations. Based on these assumptions, the sensorimotor simulation model (SMM) posits that people register information about perception and movement during encoding, and afterward, when recalling encoded events, these representations are reactivated. It is therefore believed that memory is the partial (or covert) reenactment of sensory, motor, and introspective states, rather than the amodal redescription of these states as suggested by digital computer-inspired theories of mind [46]. By arguing that the processes that encode information are also stored in memory and could be used to speed up retrieval, Kolers [47] anticipated the SMM, thus making it impossible to track a clear distinction between storage and processing.

The paragraph discusses BM as a phenomenon where past bodily experiences resurface in the present, enhancing the quality of experience. Drawing from phenomenology, it distinguishes between implicit and non-conscious memory and explicit and conscious memory. BM is tied to embodiment [6], shaping individual uniqueness by implementing past experiences without making them explicitly present. It is portrayed as a Gestalt between the brain and the body, influencing perception and movement styles. Researchers, such as Fuchs and Kolter, explore BM’s connection to trauma and pain, often associated with negative experiences. Riva’s model of BM encompasses various body representations developed over a person’s lifetime, closely tied to self-development. The body is highlighted as the most important object in the surrounding space, with specific neural areas processing body-related information. 

## 4. Research and Experimental Tasks on Body Memory

Considering that several articles using the phenomenology perspective don’t measure phenomena but try to describe them, an important problem for the cognitive sciences is how to measure and operationalize BM [14,48,49,50]. The connection between implicit and explicit memory is useful for this purpose. An interesting viewpoint by Fuchs [6] affirmed that some movements or perceptions of our body may take us back to a past situation where we experienced similar bodily sensations or perceptions through a bodily resemblance that occurs spontaneously and not through explicit memory. This is the case, for example, of *déjà-vu experiences*, where one has the sensation of reliving an experience that has already taken place in exactly the same way: past and present bodily experiences overlap and coexist. It could therefore be useful to study the experiences of déjàvu to access a greater knowledge of the memory of the body.

Another opportunity to investigate BM could be to *study embodied behavior styles*. These behavioral patterns reflect and are influenced by the intricate interplay between neural activities and the body or, in other words, represent how people act and behave in the physical world, influenced by their bodily, emotional, and cognitive experiences. Through videotapes [6] showing people walking, gesturing, moving in their environment and behaving in a certain way, it could be possible to observe congruences between body behavior and their personality characteristics [6]. When we revisit a situation, it is easier to remember itselements or components because they are part of the embodied situation that we can recall. Autobiographical memories [51] can also be considered a form of sensorimotor simulation, an embodied model of the original event through which people relive the same visual, kinesthetic, spatial and emotional information of a given past experience. Dijkstra, Kaschak, and Zwaan [52], for example, proposed an experiment to study how body posture can affect the retrieval of autobiographical memories. They believe that assuming the same body posture as during the original experience, rather than an incongruent posture, can make it easier to recall memories. In their study, they asked participants to retrieve autobiographical memories of specific past events while assuming different body positions that could be either congruent (e.g., remaining lying down on a recliner while remembering the last dentist visit) or incongruent (e.g., remaining lying down on a recliner while remembering the last football match) with the original one. The results showed that recalling memories was faster and more accurate when the body position was congruent with the memory. Even after two weeks, participants were better able to retrieve memories in the congruent condition compared to the incongruent one. In essence, having a body position congruent with the memory can help people access their memories more easily [52].

In addition to posture, memory traces contain a lot of information about a person’s movements and position when they experience something. It is thought that this same sensorimotor information is triggered again when the person tries to recall the episode. Based on the relationship between body and memory processes, two predictions have been made:

(1) Recreating the same processes used during the encoding phase (for example, in terms of body posture, position in space, etc.) can help improve memory retrieval. If memories are simulations that reconstruct the original event along with its sensorimotor components, then activating those components during recall should help speed up the retrieval process [53].
(2) Tasks involving the same neural resources as those involved during recall are expected to slow down the retrieval process. Essentially, the sensorimotor simulation may be hindered by another task that engages the same sensorimotor resources [54].

Various experimental settings have evaluated these aspects, focusing on the impact of *eye movements, co-speech gestures, body postures and movements, and bodily expressions of emotions* on cognitive processes. For instance, it has been observed that there is a high correlation between gaze patterns during perception and recall [55]: eye movements seem to play a functional role in the retrieval process, suggesting that they may imply a reenactment of the initial experience. Laeng, Bloem, D’Ascenzo, and Tommasi [56] conducted three experiments where participants first examined a stimulus and were then prompted to recall it, i.e., to study the role of eye movement in recall. In the first experiment, they found that mentally visualizing previously observed objects (such as plain triangles with different orientations and forms) led to a pattern of eye fixations that replicated the shape and orientation of each object. In the second experiment, they observed that during recall, eye movements mirrored those used to observe the objects in the initial phase of the study, even when more elaborate shapes were employed. Such mirroring predicted accuracy in memory tasks, and participants who reproduced eye movements during recall closely resembling the original movements also exhibited higher scores in spatial memory tasks. In the last experiment, memory performance was significantly reduced when the gaze was compelled to remain fixed on a point distant from the original fixations, indicating that disrupting the gaze during recall diminishes the quality of memory.

Furthermore, gestures of hands and arms often accompany speech and are intertwined with verbal content [57,58,59]. Depending on the context, gestures can add information and disambiguate intentions, thus playing a significant communicative role [60]. However, gestures are not exclusively communicative; people also tend to gesture in contexts where their interlocutors cannot observe them (for example, during a phone call [61]). Even congenital blind people use gestures, even when consciously speaking to a blind listener (see, for example [62]). It has been observed that arm gestures have roles beyond communication [63] and indirectly facilitate the maintenance of spatial representations in working memory. Through feedback from effectors or motor commands, they also directly activate the sensorimotor information that is part of mental representations. In a study by Wesp, Hesse, Keutmann, and Wheaton [64], participants who were asked to describe a painting from memory (vs. presented visually) exhibited more gestures when compared to when spatial information was visually available. This indicates that gestures helped participants by supporting the spatial and motor information associated with stored mental representations [64]. Moreover, it has been highlighted that gestures can also influence and shape mental simulations. From this perspective, gestures can both reflect and trigger a “sensorimotor simulation” [54], inducing a beneficial effect in terms of learning and memory, both during the encoding and retrieval phases.

Since a stimulus is stored in the motor pathways involved when that stimulus is initially processed, gestures during encoding can strengthen these motor pathways, and during retrieval, they can facilitate their reactivation. Supporting this idea, it has been found that memory is enhanced when items to be remembered are accompanied by gestures during encoding and when participants observe a speaker gesturing while pronouncing the items (e.g., [65,66]). Cook, Yip, and Goldin-Meadow [67] presented, for example, participants with a series of short vignettes, asking them to provide detailed descriptions. They found that, after a shot delay or a 3-week delay, recalling the vignettes linked to gesturing during their initial description resulted in higher recall rates, regardless of the duration of the delay. In a subsequent experiment, memory performance was improved even when participants were explicitly asked to gesture or not, rather than being permitted to do it spontaneously [67].

Moreover, different studies have revealed that non-verbal expressions, positive or negative, can influence memory retrieval. For instance, remembering an autobiographical event when smiling and in an upright posture can enhance positive memories [68]. In a more recent study [69], participants were manipulated using biofeedback to alter their walking patterns to mimic either depressed or happy walking styles. They then encoded and recalled emotionally loaded terms while walking. Participants who walked in a depressed style showed less difference between positive and negative word recall compared to those who walked in a happy style, indicating that walking style impacts memory functions. In brief, altering somatic states can influence the emotional components that come with specific memories; therefore, the body and its morphology have a significant impact on the processing of emotional information [70]. These results demonstrate the significance of sensory and motor functions, as well as affective valence, in the process of memory retrieval. They align with the viewpoint that regards cognitive processes as inseparable components of the sensorimotor environment in which they exist. Bodily experience extends beyond an emotional state that exceeds a specific threshold; it is an integral aspect of that emotional state. Then, it is possible to deduce that manipulation of bodily expressions could, in turn, influence mood. From a clinical perspective, this entails exploring whether individuals suffering from specific conditions like depression or other mood disorders, characterized by pessimistic thoughts and heightened recollection of negative experiences, could find relief through the manipulation of body posture and movements (see, for example, [71]).

This paragraph discusses the importance of eye movements, non-verbal expressions, body postures and gestures in the learning and memory process according to the embodied cognition framework. Eye movements replicate initial experiences during recall, and gestures facilitate spatial and motor information associated with mental representations. Gestures can also influence and shape mental simulations, contributing to the facilitation of learning and memory during the encoding and retrieval phases. Also, *incorporated behavioral styles* and *déjà-vu experiences* can allow access to BM. All these processes and body signs can be an initial way to measure and operationalize BM, overcoming the issues of the phenomenological approach. Moreover, in parallel to another issue like consciousness, here it is difficult to think about a measure of BM because here we are not focused on the intrinsic capacity of organic material to elaborate information in a more or less complex way but we refer to the content of the information. Previous information was stored and compared to the new information, and future research is likely needed to provide new tools for measuring this capacity of comparison, like the Temporo-spatial Theory of Consciousness (TTC), which, assuming a multitude of different timescales, can take into view the temporal integration of specific phenomenal contents with other phenomenal contents over time [72].

## 5. Rehabilitation Approaches and Body Memory

Riva defines “embodied medicine” [2,7,51] as the use of advanced technology to improve our health and well-being by modifying the body matrix, a coherent body representation that combines different body inputs [73,74]. The body matrix can be damaged, malfunctioning, or altered, contributing to the development of, or could be influenced by, different neurological and psychiatric disorders that affect our body experience [75]. According to Riva [2], there are two mechanisms behind this:

(1) The inability to connect bodily signals with their potential consequences (pleasant or aversive);
(2) The impairment in the ability to use new inputs from real-time perception to update the body matrix.

Riva identifies two approaches to modifying a dysfunctional body matrix through technology:

(1) Facilitate the integration of external and inner body signals [76,77,78];
(2) Improve bodily representations by inducing a controlled discrepancy between dysfunctional content and actual sensory input [79,80].

These strategies can be summarized as interoceptive feedback and bodily illusions [7]. To put these approaches into practice, some useful tools have emerged, such as the *biofeedback technique*, like *interoceptive feedback* [78,81] or *sonoception,* which consists of the modification of internal body signals through the use of sound and vibration [77,82], and *body illusion techniques*.

*The biofeedback technique* aims to alter physiological parameters (e.g., heart rate, EEG, skin conductance) returning the physiological impulses in the form of acoustic/visual signals. In particular, *interoceptive feedback* has been used [78], for example, to improve interoceptive accuracy in patients with anxiety, by providing real-time feedback of heart-type interoceptive information through augmented reality.

An example of *sonoception* is demonstrated in a study conducted by Azevedo and colleagues [77]. The study involved wearing devices on the wrist that would send discrete vibrations, mimicking the heartbeat. This helped in regulating the levels of arousal and calmness, especially in reducing physiological arousal and subjective experience associated with public speaking anxiety.

The effectiveness of *body illusion techniques* in altering the perception of one’s body is demonstrated by several authors, for example, in a study by Serino and colleagues [83]. The study found that by inducing a body illusion that shifted the sense of ownership of one’s body to another, it altered the perception and feelings associated with one’s body. Even in obese patients, the illusion helped in reducing body size distortions and decreasing dissatisfaction with one’s body.

To access BM, it is possible to focus on movements, as occurs in movement therapies that emphasize sensory and movement pathways work on both painful and traumatic as well as joyful memory contents (e.g., [84]). Examples of movement therapies include Functional Relaxation [85] and Focusing (e.g., [86,87]). Gendlin defined bodily memory as pre-reflexive and preverbal reactions of the body towards a particular situation called ‘felt sense’ [88]. Body therapy mainly works on these pre-verbal experiences, making them explicit and allowing interaction between pre-reflective and reflective aspects of the movement process. Verbalizing the experience enhances understanding of its meaning and changes the feelings it invokes [6].

*Movement therapies* can help treat trauma by allowing individuals to re-enact painful experiences to face them and gain a new perspective on life [84,89]. BM also plays a role in treating dementia [90], where implicit memory remains intact while explicit memory is lost. Embodied therapies, which work on stored bodily memories, can promote resilience, positive emotions, and contentment. Two approaches to this way are “interoceptive exposure therapy” and training people to become more sensitive to different interoceptive sensations [91]. Also, mental techniques can control and modify bodily perceptions. For instance, *autosuggestion* and *autogenic training* can modulate tactile and painful bodily experiences, such as somatic symptoms, by allowing individuals to overwrite erroneous perceptions through the reiteration of an idea, e.g., “my arm is warm” instead of the physiological state “my arm hurts” [92].

Virtual reality (VR) is another promising technique in the study of BM because it allows us to reactivate and modify traces of stored memory. It is an embedded technology that can create realistic, controlled, and individualized environments, particularly indicated for the treatment of some mental disorders [93], which was used previously in the study of spatial navigation [94] and emotional experiences [95]. It is a useful tool to induce body illusion, altering the sense of embodiment, which involves the sense of being the owner of the body (body ownership), the sense of being located in a specific place (self-location), and the sense of being the subject who perform the motor command to make a specific action (agency). Body illusion techniques are then capable of altering both perceptual and motor processes, allowing a wide range of applications in clinical settings.

**Figure 1:**
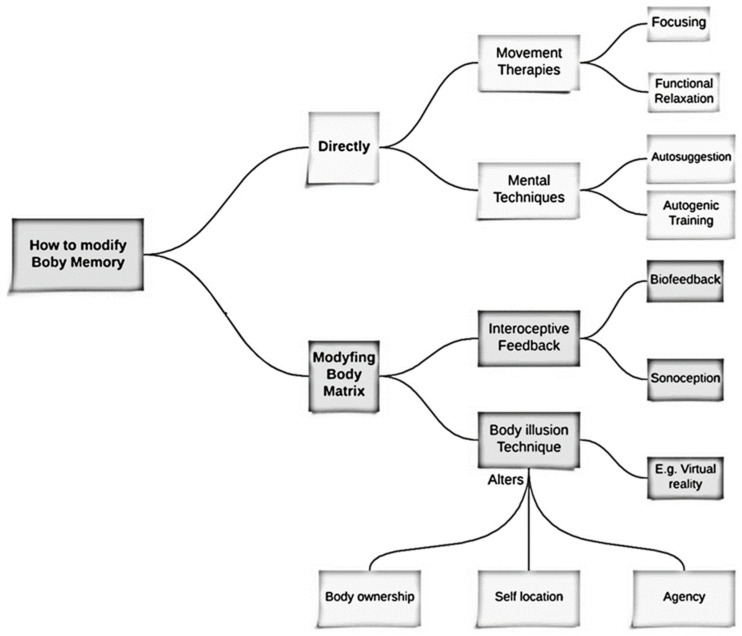
Comprehensive conceptual map that showcases the techniques/therapies that are utilized to modify BM. To enhance its clarity, we have categorized them into two groups. The first group comprises techniques that directly target BM and show a beneficial effect on some clinical conditions such as trauma, dementia, and pain. The second group is focused on modifying the body matrix, which is conceived as the multisensory representation of the body and the surrounding space that forms the foundation of BM, which showsthis efficacy, for example, in the treatment of anxiety and obesity.

What characterizes and joints all these techniques is the possibility of modifying dysfunctional or “defective” body memories into functional and more adaptive body memories. Figure 1 summarizes the main concepts of this paragraph.

## 6. Discussion

The research field of body representations is rapidly developing, as demonstrated by the growing number of studies on the topic in the last two decades. In the above chapters, we have presented an overview of the BM, which, together with the other concepts of body schema and body image, for example [5,96], represent a theme of extreme importance, especially in the field of neurological and psychiatric rehabilitation.

In contrast to the other body representations (for extensive discussions, see [5,97,98]), BM appears as one of the most faded, considering that the term “memory” itself includes different aspects (e.g., implicit vs. explicit, conscious vs. unconscious) and that this term could be considered as an umbrella term as affirmed by some authors who considered that all cognitive function is possible only in the presence of memory functions [99,100]. Therefore, in this final section, we want to discuss the evidence reported above, considering, in particular, the lens of ontogenic development of body representations.

In the previous paragraphs, we reported different viewpoints regarding the possible nature of BM. In particular, resuming the different sections, the results above reported could be divided into two macro areas: (i) how the body influences our memories and (ii) how memories, in their extensive meaning, could generate and/or influence metarepresentations (e.g., the ability to reflect on or make inferences about one’s own cognitive representations or those of others.). Regarding the first point, it is interesting to that, in terms of the models derived from Glenberg’s viewpoint [42,43], the BM is an active process of sensory-motor integration in which cognition is strongly influenced by the body. This implies that memory is directly influenced by the body and that all memories are mediated through the body rather than solely being a mere recollection of past encoded events. Considering that we cannot have experiences without our body, it could be interesting in the future to analyze the difference in BM in particular settings, for example, manipulating the objects (obtained with or without movements) or part of the body (BM in subjects immediately after an amputation due to the trauma of surgery) to study the existence and, if affirmative, the nature of BM.

The second point encompasses a diverse array of interrelated sensory and movement abilities essential for multiple purposes [101], including the perception and localization of somatic stimuli, action planning, and body awareness. These functions are distinct from each other and regulated by different brain circuits, although not completely independent. Recently, it has been suggested that our bodily experience is constructed from early development through the continuous integration of sensory, social, and cultural data from the six different representations of the body conceptualized by Riva and described above. In this sense, we reported as Riva affirmed that these representations can be combined in a coherent supramodal representation of the body and the space around it that integrates all sensory modalities (touch, proprioception, vision and vestibular signals) in a plastic representation of the body, called the “body matrix”. This integration is possible through predictive, multisensory processing activated by central, top-down, and attentional processes. From an evolutionary perspective, the body matrix has as its primary objective to allow the self to protect and extend its boundaries on both a homeostatic and psychological level [73,102,103]. It allows us to ensure a sense of bodily ownership over time while simultaneously allowing for a sufficiently flexible representation of our body to adapt to the changes that occur in our bodies throughout life. In this view, the body matrix allows the resolution of potential conflicts between body representations by producing a coherent representation of the body and the surrounding space based on their contents and here became fundamental as the term memory could be the red thread for self-generation. Specifically, through connections between the posterior parietal cortex (spatial information processing and multisensory integration), the premotor cortex, and the insula (enteroceptive awareness concerning internal states of the body), the body matrix integrates data from somatotopic and peripersonal sensory systems with body-centered spatial sensory data and with data from vision and memory of object-centered body image. Through the implementation and recognition of motor patterns, the self extends its boundaries (peripersonal space) [104,105,106,107,108]. Discussing brain localization for BM poses a challenge due to the difficulty in determining the amount of information processed at the level of the central nervous system (CNS), by the peripheral systems, or directly by the musculoskeletal apparatus based on the models presented. The thalamus could potentially play a crucial role as it is one of the main systems that integrates information from multiple bodily/sensory/motor areas. This unresolved aspect will require further exploration in the future, given the complexity of the topic.

Another interesting point that linked meta-representation and BM could be intrinsically connected to the nature of BM. As we described in the introduction, it is not clear if we can talk about BM and, if so, what kind of memory we are talking about? Analyzing studies on trauma, we can perhaps argue about a kind of episodic memory strictly related to the body (damage) but other interpretations could be possible. For example, we can ask if BM is a sort of memory based on first-order maps or involves also higher representations and a metacognitive process. In other words, are BMs fundamental for body representation itself (structural viewpoint) or, for example, to know what our body is able to do (functional viewpoint)?

In conclusion, the need for an extensive model of the interaction of body representations and human actions/movements should be at the center of future research. Considering the neurorehabilitation perspective, therefore applying the neuropsychological principle of dissociation, it is possible to identify some evidence derived from case reports in which one representation is impaired while the others are not as reported in the previous chapters as well as in other research [109], (i.e., phantom limb, autoscopic phenomena, etc.) and we considered unavoidable the study of body representations in pathological settings in which is possible to study both behaviors after central nervous system lesions as well as after body lesions in a context of unaltered brain areas. 

Our phenomenological experience of the body is shaped by early egocentric representations as well as in an allocentric frame of reference, so future studies are required in clinical settings to foster the way for a neuroscientific approach to rehabilitation, developing protocols able to offer new solutions for the study and the re-habilitation of movement able to provide information about the existence of BM and its nature.

## Figures and Tables

**Table 1:** Brief definitions and implications of the different forms of BM in Fuchs’s taxonomy [15].

	Body Memory Taxonomy of Fuchs	Implications
*Habitual/procedural body memory*	It consists of patterns of movement and perception, habits and dealing with instruments and other skills that have been formed by repetition and automation, like driving a car, playing an instrument, biking, etc.	- It borders our experiential possibilities, giving shape to an **individual style of experiencing**. - Habits are contextually enacted and may be modified within that context.
*Situational/situated body memory*	It consists of the involuntary emergence of images and sensible impressions, which are mostly affectively and emotionally charged.	- It sets the sense of familiarity/foreignness with specific situations. - Re-experiencing certain features of that situations makes us assume certain **bodily attitudes** in a pre-reflective and spontaneous way.
*Intercorporeal body memory*	It is constituted by non-verbal interactions determined by earlier experience, which is implicitly and unconsciously present in every encounter.	- Motor, emotional and social skills in childhood are integrated into implicit affective-interactive schemas (see Stern [34]—“schemes of being with)”. - Those schemas influence the formation of **relational styles and individual personality**, which are reflected in habitual bodily posture in specific contexts/situations.
*Incorporative body memory*	It is based, in particular, on the “interiorization” of the gaze of the other.	- Individuals can internalize attitudes and behaviors from others by imitating and identifying with them [12]. This process lead to adopt certain poses, manners or gender roles modifying the own primary bodily schemas of expressions in relation to the social and cultural context. It then enables the development of specific bodily attitudes and the assumption of **embodied social roles**.
*Traumatic body memory*	Refers to the impact traumatic experiences have on the present.	- The traumatic event may not be appropriated and integrated into a meaningful context and a coherent self-image. - During times of extreme stress, thoughts and emotions can become disconnected from bodily and physical sensations [35]. - Traumatic memories can be triggered by specific stimuli, so our current bodily experience, such as posture, movement, and surroundings, can bring back memories, cognitions and affects related to a similar former experiences.

**Table 2:** Different representations of the body described by Riva [2] and their relationship with the evolution of the self.

Six Body Representations in Relation to the Development of the Self (From Riva)	
***Sentient Body***	An invariant spatial structure topologically defined that, starting during fetal life, integrates the signals of the interoceptive homeostatic system with pro-prioceptive and vestibular sensations. The experiential outcome of this representation is minimal phenomenal self-hood, the experience of being present in a sentient body distinguished from the external world.
***Spatial Body***	The integration in an egocentric frame of reference coming from afferent sensory information, i.e., retinal, somaesthetic, proprioceptive, vestibular, and auditory information. The experiential outcome of this representation is *self-location* (i.e., experiencing where “I” am).
***Active Body***	The integration in an egocentric frame of afferent sensory information with efferent movement’s information thanks to the visuomotor synchrony of the stimuli. It is the process of being able to perceive visual and proprioceptive information as integrated. The experiential outcome of this representation is *agency*, the experience of controlling bodily actions.
***Personal Body***	The integration of the different components of the body in a coherent whole-body representation. The experiential outcome of this representation is *whole-body ownership* (Me), the subjective experience of owning a whole body.
***Objectified Body***	A third-person representation of one’s own body. The experiential outcome of this representation is the objectified self (the sense of *mine*), the objectified sense of what belongs to the self, including the reflective experience of being exposed and visible to others. It is the precursor of the *autobiographical self*.
***Social Body***	The integration in an allocentric frame of the objectified body with social rules and narratives related to the body. It is the result of the comparison between the “actual body” and the “ideal body”, according to social rules. The experiential outcome of this representation is the body *satisfaction/dissatisfaction* generated by the reflective experience of having a body in agreement/disagreement with social norms (Ideal Me).

## Data Availability

Not applicable.
